# Association of Self-reported High-Risk Allergy History With Allergy Symptoms After COVID-19 Vaccination

**DOI:** 10.1001/jamanetworkopen.2021.31034

**Published:** 2021-10-26

**Authors:** Lily Li, Lacey B. Robinson, Rajesh Patel, Adam B. Landman, Xiaoqing Fu, Erica S. Shenoy, Dean M. Hashimoto, Aleena Banerji, Paige G. Wickner, Upeka Samarakoon, Christian M. Mancini, Yuqing Zhang, Kimberly G. Blumenthal

**Affiliations:** 1 Division of Allergy and Clinical Immunology, Department of Medicine, Brigham and Women’s Hospital, Boston, Massachusetts; 2 Harvard Medical School, Boston, Massachusetts; 3 Division of Rheumatology, Allergy and Immunology, Department of Medicine, Massachusetts General Hospital, Boston; 4 Department of Medicine, Brigham and Women’s Hospital, Boston, Massachusetts; 5 Department of Emergency Medicine, Brigham and Women’s Hospital, Boston, Massachusetts; 6 Division of Infectious Diseases, Department of Medicine, Massachusetts General Hospital, Boston; 7 Occupational Health Services, Mass General Brigham, Boston; 8 Mongan Institute, Massachusetts General Hospital, Boston

## Abstract

**Importance:**

Allergic history in individuals with confirmed anaphylaxis to a messenger RNA (mRNA) COVID-19 vaccine is common. However, the risk factors for allergy symptoms after receiving the vaccine are unknown.

**Objective:**

To assess the association between self-reported history of high-risk allergy and self-reported allergic reactions after mRNA COVID-19 vaccination of health care employees.

**Design, Setting, and Participants:**

This cohort study obtained demographic, medical, and vaccine administration data of employees of Mass General Brigham from the institutional electronic health record. Employees who received at least 1 dose of an mRNA COVID-19 vaccine between December 14, 2020, and February 1, 2021, and who completed at least 1 postvaccination symptom survey in the 3 days after vaccination were included.

**Exposures:**

Self-reported history of high-risk allergy, defined as a previous severe allergic reaction to a vaccine, an injectable medication, or other allergen.

**Main Outcomes and Measures:**

The primary outcome was 1 or more self-reported allergic reactions in the first 3 days after dose 1 or dose 2 of an mRNA COVID-19 vaccine. Multivariable log binomial regression was used to assess the association between allergic reactions and high-risk allergy status.

**Results:**

A total of 52 998 health care employees (mean [SD] age, 42 [14] years; 38 167 women [72.0%]) were included in the cohort, of whom 51 706 (97.6%) received 2 doses of an mRNA COVID-19 vaccine and 474 (0.9%) reported a history of high-risk allergy. Individuals with vs without a history of high-risk allergy reported more allergic reactions after receiving dose 1 or 2 of the vaccine (11.6% [n = 55] vs 4.7% [n = 2461]). In the adjusted model, a history of high-risk allergy was associated with an increased risk of allergic reactions (adjusted relative risk [aRR], 2.46; 95% CI, 1.92-3.16), with risk being highest for hives (aRR, 3.81; 95% CI, 2.33-6.22) and angioedema (aRR, 4.36; 95% CI, 2.52-7.54).

**Conclusions and Relevance:**

This cohort study found that self-reported history of high-risk allergy was associated with an increased risk of self-reported allergic reactions within 3 days of mRNA COVID-19 vaccination. However, reported allergy symptoms did not impede the completion of the 2-dose vaccine protocol among a cohort of eligible health care employees, supporting the overall safety of mRNA COVID-19 vaccine.

## Key Points

**Question:**

What is the association between self-reported history of high-risk allergy and allergic reactions after messenger RNA (mRNA) COVID-19 vaccination?

**Findings:**

In this cohort study of 52 998 health care employees, self-reported high-risk allergy history was associated with an increased risk of self-reported allergic reactions after mRNA COVID-19 vaccination. Most of the reported allergy symptoms, however, did not impede the completion of the 2-dose vaccine protocol.

**Meaning:**

This finding suggests that the mRNA COVID-19 vaccines are safe to receive for eligible individuals.

## Teaser

This cohort study examines the risk factors for developing a severe or an immediate allergic reaction to an messenger RNA–based COVID-19 vaccine.

## Introduction

The messenger RNA (mRNA) COVID-19 vaccines from Pfizer-BioNTech and Moderna received emergency use authorization from the US Food and Drug Administration in December 2020 for prevention of severe COVID-19. Within days of the authorization, several reports of severe allergic reactions to the mRNA COVID-19 vaccines emerged, generating widespread concern regarding vaccine safety and creating a barrier to public health mass vaccination efforts.^1^ Currently, the only contraindications for receipt of the mRNA COVID-19 vaccines are a known history of a severe or an immediate allergic reaction to any vaccine component, which includes the excipient polyethylene glycol (PEG), and an allergic reaction to a previous dose of an mRNA vaccine.^2,3^

Since December 2020, the rate of allergy symptoms that have been reported after mRNA COVID-19 vaccination has been approximately 2%,^4^ and the incidence of anaphylaxis has been estimated as 0.025 to 2.47 cases per 10 000 vaccinations.^4,5,6,7,8,9^ Among individuals with confirmed anaphylaxis to mRNA COVID-19 vaccines, about one-third reported a history of anaphylaxis.^4,7^ National and international guidelines on mRNA COVID-19 vaccination have varied and included mixed messages for individuals with a history of anaphylaxis.^10,11,12^ In the US, the Centers for Disease Control and Prevention (CDC), national task forces, and health care institutions developed prescreening tools to risk-stratify individuals according to a clinical assessment of their high-risk allergy history, including a severe allergic reaction to an injectable medication, vaccine, PEG, or anaphylaxis from any other cause.^13,14,15^ However, these recommendations were based solely on expert opinion, and whether high-risk allergy is associated with allergy symptoms after mRNA COVID-19 vaccination is unknown.

Identification of risk factors for allergy symptoms after COVID-19 vaccination will guide safe vaccination practices for individuals at the highest risk and inform strategies that target vaccine hesitancy that is associated with allergy concerns. In this cohort study, we aimed to assess the association between self-reported history of high-risk allergy and self-reported allergic reactions after mRNA COVID-19 vaccination in a large prospective cohort of health care employees.

## Methods

### Study Population and Data Sources

Mass General Brigham (formerly Partners HealthCare System) is an integrated health care system comprising 16 health care institutions in northeastern US and is the largest employer in Massachusetts with approximately 87 000 employees. We prospectively studied Mass General Brigham employees who received at least 1 dose of an mRNA COVID-19 vaccine and completed at least 1 postvaccination symptom survey assessment. This study was approved by the Mass General Brigham Institutional Review Board, which granted a waiver of informed consent because the study was secondary observational research. We followed the Strengthening the Reporting of Observational Studies in Epidemiology (STROBE) reporting guideline.^16^ The study period was from December 14, 2020, to February 1, 2021, and follow-up was conducted through March 1, 2021.

The Mass General Brigham institutional vaccination procedures and voluntary reporting of allergy symptoms after vaccination have been previously described in a study of allergic reaction and anaphylaxis incidence after mRNA dose 1 (which occurred from December 16, 2020, to February 12, 2021).^4^ Briefly, the prevaccination allergy risk assessment was performed through a screening questionnaire (eMethods in the Supplement) that was completed by the employees either online or by telephone or in person (with assistance from a hospital staff member) for those without computer or smartphone access. Completion of the prevaccination questionnaire was required of all employees, who were asked to attest that their answers were truthful.

Employees were directed via email or a hyperlink on an existing custom COVID-19 symptom-check application^17^ to record and report their own postvaccination symptoms daily for 3 days after vaccination using a web-based survey (REDCap; Vanderbilt University) (eMethods in the Supplement). For employees without computer or smartphone access, symptom checks were conducted by hospital staff by telephone call or text message. Survey completion was defined as full completion of at least 1 of the 3 postvaccine symptom surveys after receiving either dose of the 2-dose vaccine series. Overall, the symptom check response rate was 88% (85% for male and 89% for female employees).

### Exposure

Exposure status (history of high-risk allergy) was ascertained before the initial vaccine administration from each employee’s completed screening questionnaire (eMethods in the Supplement). Employees who reported no history of a severe allergic reaction received an mRNA COVID-19 vaccine with a standard 15-minute observation period. Individuals who reported any history of a severe allergic reaction to an injectable medication, vaccine, or other allergen (eg, food, venom, drug, or latex) were considered to be at higher risk and eligible for an mRNA COVID-19 vaccine with a 30-minute observation period, per CDC guidelines.^18^ Those with a self-reported history of an immediate or a severe allergic reaction to a component of the vaccine (eg, PEG or polysorbate) required evaluation with a Mass General Brigham allergist before vaccine eligibility, although ultimately few employees were found ineligible for the vaccines.^13^

### Outcomes

The primary outcome was 1 or more self-reported allergy symptoms in the first 3 days after either dose 1 or dose 2 of an mRNA COVID-19 vaccine. Reportable allergy symptoms included itching or rash (other than at the injection site), hives, angioedema (swollen lips, tongue, eyes, or face), and respiratory symptoms (wheezing, chest tightness, or shortness of breath) (eMethods in the Supplement).

The secondary outcome was self-reported severe allergy symptoms to the mRNA COVID-19 vaccine. These symptoms included (1) hives, itching, or rash other than at the injection site, and (2) either respiratory symptoms, angioedema, or both in the first 3 days after vaccination. For employees whose symptoms were considered severe, we identified the frequency with which they were evaluated by an allergy specialist at Mass General Brigham and whether the specialist determined that the symptoms were consistent with a true allergic response.

### Covariates

We obtained employee demographic data, including age, sex, and race and ethnicity (which were self-reported by employees), from the Mass General Brigham electronic health record (EHR). Race was categorized as Asian, Black, White, other (ie, American Indian, Native Hawaiian, or individuals who identified as belonging to 2 or more race categories), or unknown, whereas ethnicity was categorized as Hispanic, non-Hispanic, or unknown. Employee role was categorized as clinical (eg, health care practitioner, pharmacist, dietitian, or physical therapist), administrative (eg, human resources or marketing), support services (eg, environmental services or food services), or other (eg, sales). Among clinical staff, health care practitioners were defined as medical doctors, registered nurses, and physician assistants. Comorbidities were identified by the presence of at least 1 *International Statistical Classification of Diseases and Related Health Problems, Tenth Revision* diagnosis code^19,20,21,22,23^ in the 12 months before vaccination. We calculated Charlson comorbidity index score (range: 0-16, with higher scores indicating a more severe condition).^24,25^ Vaccine administration data, including manufacturer and Mass General Brigham satellite site, were obtained from the EHR.

### Statistical Analysis

We described the characteristics of employees according to their history of high-risk allergy. Categorical variables were compared using a χ^2^ test, and continuous variables were compared using an unpaired, 2-tailed *t-*test or Wilcoxon rank sum test, as indicated.

We estimated the risk of allergic reactions after vaccination by high-risk allergy status and examined their association using log binomial regression. In the multivariable regression model, we adjusted for demographic factors, medical comorbidities, and vaccine manufacturer as well as present adjusted relative risk (aRR) with its 95% CI as the measure of effect. Allergic comorbidities from diagnostic codes were not included in the model, as they likely represent the exposure of interest (high-risk allergy).^26^ To establish whether the association between high-risk allergy status and risk of allergic reactions after vaccination was modified by other risk factors, we examined the association of high-risk allergy status within the strata of sex, age (<55 years vs ≥55 years), race (Black, White, or other), and vaccine manufacturer. We tested for a modification of the association by including an interaction term (between high-risk allergy status and each risk factor) in the multivariate regression model.

All analyses were performed using SAS, version 9.4 (SAS Institute). Two-sided *P* < .05 was considered to be statistically significant.

## Results

A total of 52 998 Mass General Brigham employees were included in the cohort. This cohort had a mean (SD) age of 42 (14) years and was composed of 38 167 women (72.0%) and 14 831 men (28.0%). Of these employees, 51 706 (97.6%) completed the full 2-dose vaccine series and 474 (0.9%) reported a history of high-risk allergy (Figure). Baseline characteristics according to high-risk allergy status are presented in Table 1. The percentages of women (80.8% [383 of 474] vs 71.9% [37 784 of 52 524]) and Black individuals (9.5% [45 of 474] vs 4.8% [2521 of 52 524]) were higher among those with a history of high-risk allergy vs those without. In total, 47 327 employees (89.3%) had at least 1 previous encounter in the EHR before vaccination. Individuals with a history of high-risk allergy compared with those without were older (46 years vs 42 years) and had more atopic disease (16.7% vs 8.7%; *P* < .001) and a higher prevalence of anxiety (28.9% vs 23.9%; *P* = .01), hypertension (14.6% vs 10.9%; *P* = .01), and malignant neoplasm (4.0% vs 2.4%; *P* = .03). Distributions of vaccine manufacturer, vaccination site, and employee role also differed by high-risk allergy status.

**Figure.**
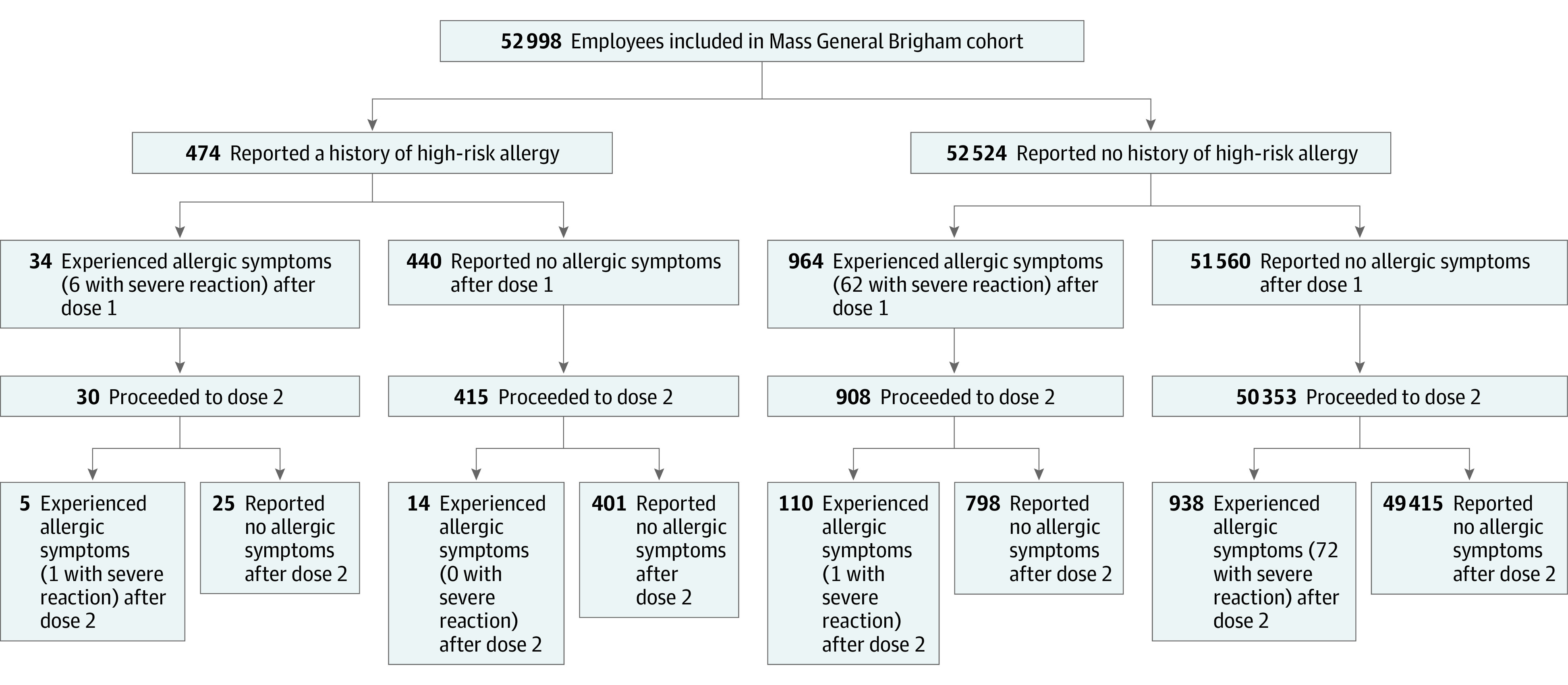
Study Cohort A total of 140 individuals reported a severe reaction after messenger RNA vaccination, including 10 individuals with reactions to both doses.

**Table 1.** Baseline Characteristics Stratified by History of High-Risk Allergy Among Employees Who Received mRNA COVID-19 Vaccine

Characteristic	No. (%)			*P* value^b^
	All employees (n = 52 998)	High-risk allergy history		
		With (n = 474)^a^	Without (n = 52 524)	
Age, mean (SD), y	42 (14)	46 (14)	42 (14)	<.001
Sex				
Female	38 167 (72.0)	383 (80.8)	37 784 (71.9)	<.001
Male	14 831 (28.0)	91 (19.2)	14 740 (28.1)	
Race				
Asian	4796 (9.0)	37 (7.8)	4759 (9.1)	<.001
Black	2566 (4.8)	45 (9.5)	2521 (4.8)	
White	35 297 (66.6)	321 (67.7)	34 976 (66.6)	
Other^c^	2810 (5.3)	28 (5.9)	2782 (5.3)	
Unknown	7529 (14.2)	43 (9.1)	7486 (14.3)	
Ethnicity				
Hispanic	2616 (4.9)	35 (7.4)	2581 (4.9)	.02
Non-Hispanic	36 009 (67.9)	328 (69.2)	35 681 (67.9)	
Unknown	14 373 (27.1)	111 (23.4)	14 262 (27.2)	
Vaccine manufacturer				
Moderna	33 247 (62.7)	216 (45.6)	33 031 (62.9)	<.001
Pfizer-BioNTech	19 751 (37.3)	258 (54.4)	19 493 (37.1)	
Vaccination site				
Massachusetts General Hospital	23 273 (43.9)	226 (47.7)	23 047 (43.9)	.002
Brigham and Women’s Hospital	13 558 (25.6)	140 (29.5)	13 418 (25.5)	
Mass General Brigham satellite site	12 881 (24.3)	82 (17.3)	12 799 (24.4)	
Newton Wellesley Hospital	3286 (6.2)	26 (5.5)	3260 (6.2)	
Employee role				
Clinical	33 571 (63.3)	215 (45.4)	33 356 (63.5)	<.001
Health care practitioner	22 566 (42.6)	131 (27.6)	22 435 (42.7)	
Administrative	9471 (17.9)	154 (32.5)	9317 (17.7)	
Research	3883 (7.3)	37 (7.8)	3846 (7.3)	
Support services	3636 (6.9)	50 (10.5)	3586 (6.8)	
Other	2437 (4.6)	18 (3.8)	2419 (4.6)	
Medical comorbidities				
Anxiety	12 678 (23.9)	137 (28.9)	12 541 (23.9)	.01
Atopic disease^d^	4670 (8.8)	79 (16.7)	4591 (8.7)	<.001
Chronic idiopathic urticaria	392 (0.7)	11 (2.3)	381 (0.7)	<.001
Hypertension	5786 (10.9)	69 (14.6)	5717 (10.9)	.01
Depression	3377 (6.4)	38 (8.0)	3339 (6.4)	.14
Diabetes	1539 (2.9)	20 (4.2)	1519 (2.9)	.09
Malignant neoplasm	1297 (2.4)	19 (4.0)	1278 (2.4)	.03
Charlson comorbidity index score, mean (SD)	0.4 (1.3)	0.6 (1.5)	0.4 (1.3)	<.001

Abbreviation: mRNA, messenger RNA.

^a^ Includes severe allergy to drug, food, latex, or venom (n = 221 [46.6%]); injectable medication or vaccine (n = 217 [45.8%]); or polyethylene glycol (n = 9 [1.9%]).

^b^ χ^2^ test except age (*t* test).

^c^ Other included American Indian, Native Hawaiian, or individuals who identified as belonging to 2 or more race categories.

^d^ Asthma, allergic rhinitis, or atopic dermatitis.

Among the 474 employees who reported a history of high-risk allergy, a past reaction to an idiopathic or known allergen (eg, food, venom, drug, or latex,) was common (n = 221 [46.6%]). A total of 217 employees (45.8%) reported a previous severe allergic reaction to an injectable medication or vaccine. Only 9 individuals (1.9%) had a history of a severe allergic reaction to PEG or PEG-containing products (eg, Miralax and injectable steroid), and 8 of these individuals were evaluated by a Mass General Brigham allergist before vaccine administration.^27^

There were 2516 of 52 998 employees (4.7%) who reported 1 or more allergy symptoms in the 3 days after receiving either dose of an mRNA vaccine (Table 2). Individuals with vs without a history of high-risk allergy reported more allergic reactions (11.6% [n = 55] vs 4.7% [n = 2461]). Mild symptoms, such as rash or itching (other than at the injection site), were the most commonly reported reaction in the cohort (1422 of 52 998 [2.7%]), followed by respiratory symptoms (687 of 52 998 [1.3%]), hives (476 of 52 998 [0.9%]), and angioedema (342 of 52 998 [0.6%]). High-risk allergy history was associated with an increased risk of allergic reactions (RR, 2.48; 95% CI, 1.93-3.18). Adjustment of other potential confounders did not change the association materially (aRR, 2.46; 95% CI, 1.92-3.16). In the adjusted analyses, a history of high-risk allergy was associated with an increased risk of hives (aRR, 3.81; 95% CI, 2.33-6.22) and angioedema (aRR, 4.36; 95% CI, 2.52-7.54) after either dose, although risks were consistently higher after dose 1.

**Table 2.** Association of History of High-Risk Allergy With Self-reported Allergic Reactions Over 3 Days After mRNA COVID-19 Vaccination

Allergic reaction	High-risk allergy history, No. (%)		Relative risk (95% CI)	
	With (n = 474)	Without (n = 52 524)	Unadjusted	Adjusted^a^
**Either dose 1 or dose 2** ^b^				
No. (%)	55 (11.6)	2461 (4.7)	2.48 (1.93-3.18)	2.46 (1.92-3.16)
Itching or rash^c^	32 (6.8)	1390 (2.7)	2.55 (1.82-3.58)	2.46 (1.76-3.45)
Hives	16 (3.4)	460 (0.9)	3.85 (2.36-6.29)	3.81 (2.33-6.22)
Respiratory symptoms^d^	10 (2.1)	677 (1.3)	1.64 (0.88-3.04)	1.76 (0.95-3.27)
Angioedema	13 (2.7)	329 (0.6)	4.38 (2.53-7.56)	4.36 (2.52-7.54)
**Dose 1** ^e^				
No. (%)	34 (7.2)	964 (1.8)	3.91 (2.81-5.44)	3.66 (2.63-5.10)
Itching or rash^c^	22 (4.6)	714 (1.4)	3.41 (2.26-5.17)	3.16 (2.09-4.79)
Hives	11 (2.3)	211 (0.4)	5.78 (3.17-10.52)	5.47 (3.00-9.99)
Respiratory symptoms^d^	9 (1.9)	306 (0.6)	3.26 (1.69-6.28)	3.24 (1.68-6.26)
Angioedema	10 (2.1)	169 (0.3)	6.56 (3.49-12.33)	6.33 (3.35-11.95)
**Dose 2** ^f^ ^,^ ^g^				
No. (%)	19 (4.3)	1048 (2.0)	2.09 (1.34-3.26)	2.10 (1.35-3.28)
Itching or rash^c^	14 (3.2)	755 (1.5)	2.14 (1.27-3.59)	2.15 (1.28-3.62)
Hives	6 (1.4)	258 (0.5)	2.68 (1.20-5.99)	2.81 (1.26-6.28)
Respiratory symptoms^d^	3 (0.7)	385 (0.8)	0.90 (0.29-2.78)	1.05 (0.34-3.25)
Angioedema	3 (0.7)	170 (0.3)	2.03 (0.65-6.34)	2.15 (0.69-6.72)

Abbreviation: mRNA, messenger RNA.

^a^ Adjusted for sex, age, race, vaccine manufacturer, and Charlson comorbidity index score.

^b^ n = 140 with reported severe allergic reaction, including 6 with high-risk allergy history. Severe allergic reaction was defined as hives or rash plus respiratory symptoms and/or angioedema.

^c^ Itching or rash was specified as other than injection site.

^d^ Respiratory symptoms included wheezing, chest tightness, or shortness of breath (eMethods in the Supplement).

^e^ n = 68 with reported severe allergic reaction, including 6 with high-risk allergy history.

^f^ n = 445 with reported high-risk allergy, and n = 51 261 with no high-risk allergy history who received both doses of mRNA COVID-19 vaccine.

^g^ n = 74 with reported severe allergic reaction, including 1 with high-risk allergy history.

The relative associations of high-risk allergy with risk of allergic reactions were consistent across strata of other risk factors, including sex, age, race, and vaccine manufacturer (Table 3). Older age (RR, 1.00; 95% CI, 0.99-1.00), Black race (RR, 1.69; 95% CI, 1.47-1.95), higher Charlson comorbidity index score (RR, 1.04; 95% CI, 1.01-1.06), and receipt of Moderna (vs Pfizer-BioNTech) vaccine (RR, 1.49; 95% CI, 1.37-1.63) were associated with an increased risk of allergic reactions after an mRNA COVID-19 vaccination, whereas male (vs female) sex was associated with decreased risk (RR, 0.66; 95% CI, 0.60-0.73) (eTable 1 in the Supplement).

**Table 3.** Self-reported History of High-Risk Allergy and Risk of an Allergic Reaction Within 3 Days After mRNA COVID-19 Vaccination

Allergic reaction	High-risk allergy history, No. (%)		Adjusted relative risk (95% CI)^**a**^	*P* value for interaction
	With	Without		
Either dose 1 or dose 2 by sex				
Male	7 (7.7)	505 (3.4)	2.50 (1.27-5.32)	.85
Female	48 (12.5)	1956 (5.2)	2.40 (1.91-3.26)	
Either dose 1 or dose 2 by age, y				
≥55	14 (9.2)	478 (4.1)	2.24 (1.35-3.72)	.60
<55	41 (12.8)	1983 (4.9)	2.63 (1.97-3.51)	
Either dose 1 or dose 2 by race				
Black	6 (13.3)	190 (7.5)	1.94 (0.91-4.13)	.62
White	34 (10.6)	1495 (4.3)	2.59 (1.88-3.57)	
Other^b^	15 (13.9)	776 (5.2)	2.59 (1.62-4.14)	
Either dose 1 or dose 2 by vaccine manufacturer				
Moderna	33 (15.3)	1764 (5.3)	2.80 (2.04-3.84)	.49
Pfizer Bio-NTech	22 (8.5)	697 (3.6)	2.30 (1.53-3.45)	

Abbreviation: mRNA, messenger RNA.

^a^ Log binomial model was adjusted for sex, age, race, vaccine manufacturer, and Charlson comorbidity index score.

^b^ Other category included American Indian, Native Hawaiian, or individuals who identified as belonging to 2 or more race categories.

Self-reported severe allergic reactions in the 3 days after COVID-19 vaccination were uncommon (140 of 52 998 [0.3%]). Of the 140 individuals who reported severe allergy symptoms after either vaccine dose, 6 (4.3%) had a history of high-risk allergy. Manual validation identified that of 65 individuals with severe reactions and who were seen by a Mass General Brigham allergist or immunologist, 41 (63.1%) had a confirmed allergic reaction. Those with reactions that were deemed nonallergic had symptoms, such as dizziness, fever, chills, tachycardia, and headache. The risk of self-reported severe allergic reactions in the 3 days after vaccination was 1.3% among employees with a high-risk allergy history and 0.3% among those without such a history. High-risk allergy history was associated with an increased risk of severe allergic reactions after mRNA COVID-19 vaccination (RR, 4.96 [95% CI, 2.20-11.19]; aRR, 5.20 [95% CI, 2.30-11.75]).

To assess whether potential misclassification of self-reported allergy symptoms after vaccination may affect these findings, we evaluated the association of high-risk allergy with the risk of allergic reactions after vaccination among clinical health care practitioners. The effect size estimates for health care practitioners were slightly higher than those for the general employee cohort, particularly after dose 1 (eTable 2 in the Supplement). In an adjusted model, a history of high-risk allergy was associated with an increased risk of allergic reactions (aRR, 2.93; 95% CI, 1.82-4.73), with the highest relative risk for hives (aRR, 7.39; 95% CI, 3.33-16.40) and angioedema (aRR, 4.76; 95% CI, 1.53-14.82).

## Discussion

In this large prospective study of health care employees in the US, 97.6% of the cohort successfully completed the recommended 2-dose mRNA COVID-19 vaccine series. We found that a self-reported history of high-risk allergy was associated with a 2.5-fold higher risk for self-reported allergic reactions in the 3 days after vaccination and an approximately 4-fold higher risk of hives and angioedema specifically. Although reporting of severe allergic reactions to an mRNA COVID-19 vaccine was rare (0.3%), those with a history of high-risk allergy were at a nearly 5-fold increased risk of these reactions, and such reactions were verified by specialists in most individuals who sought clinical care for their symptoms within the Mass General Brigham system. These identified associations were not sensitive to covariate adjustment and persisted across subgroups that were stratified by these factors.

Severe allergic reactions to vaccines are rare, with a reported incidence of 1.31 to 7.91 cases per million vaccine doses.^9,28^ However, a recent study of 429 highly allergic individuals who received the Pfizer-BioNTech vaccine under medical observation identified a much higher rate of minor allergic reactions (1.4%) and anaphylaxis (0.7%).^29^ Past vaccine reactions have largely been attributed to excipients rather than to vaccine active ingredients.^13,30^ The cohort in the present study included 217 employees with a history of a severe allergic reaction to an injectable medication or a vaccine and 9 employees with a history of severe allergic reaction to PEG. By reported history alone, many of these individuals may have been ineligible for an mRNA COVID-19 vaccine, according to many international guidelines. However, following the CDC guidelines, with allergist consultation, risk stratification, and shared decision-making, all employees were able to complete the 2-dose vaccine series.^27^ For the rare individuals with a history of severe allergic reactions to PEG, consultation with an allergist or immunologist is recommended because the mRNA vaccine may not be an absolute contraindication for such individuals.

Lessons may also be learned from previous restrictions of vaccine based on excipient allergy. For example, concerns were raised regarding the safety of administering egg-containing immunizations to individuals with an egg allergy during the H1N1 influenza A pandemic from 2009 to 2010 and played a role in global vaccine hesitancy. A large body of evidence subsequently emerged that indicated inactivated influenza vaccine to be safe for those with an egg allergy, and current national and international guidelines now recommend the administration of influenza vaccine to individuals with an egg allergy of any severity.^31^ Future studies involving diverse populations are needed to help address ongoing questions regarding the safety of mRNA COVID-19 vaccines in individuals with suspected or confirmed PEG allergy. Moreover, the emergence of allergy symptoms on first exposure, as was more commonly observed in this study, is atypical for IgE-mediated reactions,^1^ and an investigation of other mechanisms (eg, non-IgE-mediated mast cell activation or complement activation) underlying these reactions is warranted.

Of individuals with allergist-confirmed severe allergic reactions to mRNA COVID-19 vaccine to date, roughly one-third reported a history of anaphylaxis and most were women.^4,7^ Cutaneous findings about Moderna arm, a delayed-onset localized skin reaction, have been described largely in female patients.^32^ In a study of more than 400 dermatological reactions after COVID-19 vaccination, 90% of participants were women in an international dermatology registry.^33^ Similarly, we found female sex to be an independent factor in self-reported allergic reactions after mRNA COVID-19 vaccination in an adjusted multivariable model. This prospective study design vastly reduces the impact of reporter bias that is observed in case series and registry studies. Further studies are needed to better understand the risk factors, including sex, for allergic reactions after receipt of an mRNA COVID-19 vaccine. A multicenter, phase 2 randomized clinical trial is currently being conducted to assess COVID-19 vaccination reactions in individuals with a history of high-risk allergy and control participants with no atopic history.^34^

Assessment of allergy symptoms after vaccination was based on self-reported reactions. Of the 175 possible severe allergic reactions in a CDC report, 86 (49%) were ultimately deemed nonanaphylactic allergic reactions.^7^ The true incidence of postvaccination allergic reactions may therefore be lower than that reported in this study. Given the scale of the national mass vaccination efforts and the volume of employees who were vaccinated in a short time frame, manual review of all reported allergy symptoms was not feasible. However, allergic reactions were validated in more than 60% of those who reported severe reactions and who had a specialist visit, and in a subgroup analysis of clinical professionals with medical knowledge and training, allowing for high-quality self-reported health data,^35^ we observed a stronger association between high-risk allergy and risk of allergy symptoms after vaccination, suggesting less random misclassification of the outcome. Although additional prospective studies are needed to further examine the risk factors for confirmed allergic reactions after COVID-19 vaccination, perceived (ie, self-reported) allergy symptoms are also critical to study because of their equivalent association with public perception and vaccine hesitancy.

Recent data indicated that, even for individuals who reported immediate and potentially allergic reactions after the first dose of an mRNA COVID-19 vaccine, the second dose can be safely administered.^36^ Similarly, in this cohort, few individuals did not complete the 2-dose vaccine regimen, raising the possibility that not all first-dose reactions are truly allergic or may occur through non–IgE-mediated mechanisms. Reasons for incomplete vaccination vary and may include ineligibility for dose 2 after an allergy evaluation, scheduling factors, or other personal reasons for vaccine hesitancy. Future work directed at better understanding the reasons for delaying or not completing COVID-19 vaccination is warranted.

### Strengths and Limitations

This study has some strengths. First, it included a large sample size; used comprehensive, prospectively collected data on allergy history (given that reporting was a requirement for vaccine eligibility); and captured more than 88% of the entire Mass General Brigham employee population with postvaccination symptom surveys. Second, we performed subgroup analyses of clinical health care practitioners and manual EHR reviews for employees who met the definition of experiencing a severe allergic reaction.

This study also has some limitations. First, the cohort consisted only of health care employees in the northeastern US, and thus the study findings may not be generalizable to health care employees in other parts of the country or to international populations.^37^ However, given the thousands of individuals who were included in this study, the findings remain informative and highly relevant as mRNA booster vaccinations begin. Second, comorbidity information may be incomplete for some individuals because they may have medical practitioners outside of the Mass General Brigham system and therefore their medical history may not be fully recorded in the EHR. However, this problem is inherent in the use of EHR data for clinical research; before comorbidity ascertainment, we identified that more than 89% of employees had at least 1 encounter that allowed for the near-complete capture of comorbidity data in the year before COVID-19 vaccination. Moreover, because self-reporting a history of high-risk allergy was required for vaccine eligibility and scheduling, per institutional protocols, exposure data were complete for all study participants. Third, adjustment for potential confounders in the multivariable models did not substantially change the effect size estimates, suggesting that any residual confounding is unlikely to jeopardize the internal validity of the findings.

## Conclusions

This cohort study found that a self-reported history of high-risk allergy was associated with an increased risk of self-reported allergic reactions after mRNA COVID-19 vaccination, but most of the reported allergy symptoms did not impede the completion of the 2-dose mRNA COVID-19 vaccine series. Risks were higher after dose 1, and the reactions with the highest risk were hives and angioedema. Severe allergic reactions were rare. This study not only highlighted that high-risk allergy history was associated with allergy symptoms after COVID-19 vaccination but also supported the overall safety of mRNA vaccines in all eligible individuals.

## Notes

Supplement. **eMethods.** Employee Pre-vaccination Screening Questionnaire and Employee Symptom Survey **eTable 1.** Predictors of Self-Reported Allergic Reactions Over Three Days Following Either Dose of mRNA COVID-19 Vaccine **eTable 2.** Association of High-Risk Allergy History With Self-Reported Allergic Reactions Over Three Days Following mRNA COVID-19 Vaccine in Subgroup of Clinical Healthcare Employees
