# Supplementary material for: Association of Self-reported High-Risk Allergy History With Allergy Symptoms After COVID-19 Vaccination
Source: JAMA Netw Open. 2021 Oct 26;4(10):e2131034. doi: 10.1001/jamanetworkopen.2021.31034 (PMC8548941; doi:10.1001/jamanetworkopen.2021.31034)
Supplement: Supplement. — eMethods. Employee Pre-vaccination Screening Questionnaire and Employee Symptom Survey eTable 1. Predictors of Self-Reported Allergic Reactions Over Three Days Following Either Dose of mRNA COVID-19 Vaccine eTable 2. Association of High-Risk Allergy History With Self-Reported Allergic Reactions Over Three Days Following mRNA COVID-19 Vaccine in Subgroup of Clinical Healthcare Employees [file jamanetwopen-e2131034-s001.pdf]

Supplementary Online Content

Li L, Robinson LB, Patel R, et al. Association of self-reported high-risk allergy history with allergy symptoms after COVID-19 vaccination. *JAMA Netw Open*. 2021;4(10):e2131034. doi:10.1001/jamanetworkopen.2021.31034

eMethods. Employee Pre-vaccination Screening Questionnaire and Employee Symptom Survey

eTable 1. Predictors of Self-Reported Allergic Reactions Over Three Days Following Either Dose of mRNA COVID-19 Vaccine

eTable 2. Association of High-Risk Allergy History With Self-Reported Allergic Reactions Over Three Days Following mRNA COVID-19 Vaccine in Subgroup of Clinical Healthcare Employees

This supplementary material has been provided by the authors to give readers additional information about their work.

© 2021 Li L et al. *JAMA Network Open.*

eMethods. Employee Pre-vaccination Screening Questionnaire and Employee Symptom Survey

Employee Pre-vaccination Screening Questionnaire – Allergy Questions

Version 1. December 14, 2020 through January 19, 2021

1. Do you have a history of a severe allergic reaction to a vaccine OR an injectable medication?
2. Do you have a history of a severe allergic reaction to polyethylene glycol?

Version 2. January 20, 2021 onwards

1. Do you have a history of a severe allergic reaction to an injectable medication (intravenous, intramuscular, or subcutaneous)?
2. Do you have a history of a severe allergic reaction to a previous vaccine?
3. Do you have a history of a severe allergic reaction to another allergen (eg, food, venom, or latex)?
4. Do you have a history of an immediate or severe allergic reaction to PEG-, a polysorbate-, or polyoxyl 35 castor oil (eg, paclitaxel)-containing injectable or vaccine?

Employee Symptom Survey—Allergy Question

Version 1. December 17 through December 31, 2020

Have you had any of the following allergic symptoms over the past day? (check all that apply)

o Rash or itching (other than at injection site)
o Hives
o Swollen lips, tongue, eyes, or face
o Wheezing, chest tightness or shortness of breath
o None of the above

Version 2. January 1, 2021 onwards

Over the past day, have you had any of the following allergic symptoms? (check all that apply)

o Rash or itching (other than where you got your shot)
o Hives (itchy, raised bumps that can look like mosquito bites)
o Swollen lips, tongue, eyes, or face
o Wheezing, chest tightness or shortness of breath that has continued since your shot
o None of the above

© 2021 Li L et al. *JAMA Network Open.*

eTable 1. Predictors of Self-Reported Allergic Reactions Over Three Days Following Either Dose of mRNA COVID-19 Vaccine

	**Unadjusted Relative Risk (95% Confidence Interval)**	**P value**	**Adjusted Relative Risk (95% Confidence Interval)**	**P value**
Sex (male vs female)	0.66 (0.60, 0.73)	< 0.001	0.67 (0.60, 0.73)	< 0.001
Age	1.00 (0.99, 1.00)	0.02	1.00 (0.99, 1.00)	0.01
Race (White)	0.77 (0.71, 0.83)	< 0.001	0.99 (0.87, 1.12)	0.83
Race (Black)	1.69 (1.47, 1.95)	< 0.001	1.77 (1.48, 2.11)	< 0.001
Vaccine Manufacturer (Moderna vs Pfizer-BioNTech)	1.49 (1.37, 1.63)	< 0.001	1.49 (1.37, 1.63)	< 0.001
Charlson co-morbidity index	1.04 (1.01, 1.06)	0.01	1.03 (1.00, 1.06)	0.02

© 2021 Li L et al. *JAMA Network Open.*

eTable 2. Association of High-Risk Allergy History With Self-Reported Allergic Reactions Over Three Days Following mRNA COVID-19 Vaccine in Subgroup of Clinical Healthcare Employees

	**High-risk allergy history** (n=131)	**No high-risk allergy history** (n=22,435)	**Unadjusted Relative Risk** (95% Confidence Interval)	**Adjusted Relative Risk[a]** (95% Confidence Interval)
**Allergic reaction -Either Dose 1 or Dose 2[b], No. (%)**	15 (11.5)	836 (3.7)	3.07 (1.90, 4.97)	2.93 (1.82, 4.73)
Itching or rash	10 (7.6)	529 (2.4)	3.24 (1.77, 5.91)	3.05 (1.68, 5.57)
Hives	6 (4.6)	138 (0.6)	7.45 (3.35, 16.56)	7.39 (3.33, 16.40)
Respiratory symptoms	2 (1.5)	179 (0.8)	1.91 (0.48, 7.63)	1.88 (0.47, 7.50)
Angioedema	3 (2.3)	104 (0.5)	4.94 (1.59, 15.37)	4.76 (1.53, 14.82)
**Allergic reaction -Dose 1[c], No. (%)**	10 (7.6)	309 (1.4)	5.54 (3.02, 10.16)	5.21 (2.85, 9.55)
Itching or rash	6 (4.6)	250 (1.1)	4.11 (1.86, 9.07)	3.84 (1.74, 8.47)
Hives	4 (3.1)	51 (0.2)	13.43 (4.93, 36.63)	13.42 (4.90, 36.74)
Respiratory symptoms	2 (1.5)	86 (0.4)	3.98 (0.99, 16.01)	3.76 (0.94, 15.08)
Angioedema	3 (2.3)	49 (0.2)	10.49 (3.31, 33.21)	9.62 (3.03, 30.55)
**Allergic reaction -Dose 2[de], No. (%)**	5 (4.0)	408 (1.9)	2.16 (0.91, 5.13)	2.04 (0.86, 4.83)
Itching or rash	5 (4.0)	310 (1.4)	2.85 (1.20, 6.77)	2.67 (1.12, 6.33)
Hives	2 (1.6)	90 (0.4)	3.92 (0.98, 15.75)	3.81 (0.95, 15.29)
Respiratory symptoms	1 (0.8)	96 (0.4)	1.84 (0.26, 13.08)	1.92 (0.27, 13.66)
Angioedema	0 (0)	55 (0.3)	..	..

[a]Adjusted for sex, age, race, vaccine manufacturer, Charlson co-morbidity index
[b]n=40 with a reported severe allergic reaction (including 2 with high-risk allergy history); severe allergic reaction defined as hives or rash, plus respiratory symptoms and/or angioedema
[c]n=21 with a reported severe allergic reaction (2 with high-risk allergy history)
[d]n=125 with reported high-risk allergy; n=22062 with no high-risk allergy history who received both doses of mRNA COVID-19 vaccine
[e]n=20 with a reported severe allergic reaction (1 with high-risk allergy history)
Itching or rash was specified as other than injection site; Respiratory symptoms included wheezing, chest tightness or shortness of breath (see eMethods in the Supplement).
Abbreviations: No., number

© 2021 Li L et al. *JAMA Network Open.*
